# The role of feedback control mechanisms on the establishment of oscillatory regimes in the Ras/cAMP/PKA pathway in *S. cerevisiae*

**DOI:** 10.1186/1687-4153-2012-10

**Published:** 2012-07-20

**Authors:** Daniela Besozzi, Paolo Cazzaniga, Dario Pescini, Giancarlo Mauri, Sonia Colombo, Enzo Martegani

**Affiliations:** 1 Università degli Studi di Milano, Dipartimento di Informatica, Via Comelico 39, 20135 Milano, Italy; 2 Università degli Studi di Bergamo, Dipartimento di Scienze della Persona, Piazzale S. Agostino 2, 24129 Bergamo, Italy; 3 Università degli Studi di Milano-Bicocca, Dipartimento di Statistica, Via Bicocca degli Arcimboldi 8, 20126 Milano, Italy; 4 Università degli Studi di Milano-Bicocca, Dipartimento di Informatica, Sistemistica e Comunicazione, Viale Sarca 336, 20126 Milano, Italy; 5 Università degli Studi di Milano-Bicocca, Dipartimento di Biotecnologie e Bioscienze, Piazza della Scienza 2, 20126 Milano, Italy

## Abstract

In the yeast *Saccharomyces cerevisiae*, the Ras/cAMP/PKA pathway is involved in the regulation of cell growth and proliferation in response to nutritional sensing and stress conditions. The pathway is tightly regulated by multiple feedback loops, exerted by the protein kinase A (PKA) on a few pivotal components of the pathway. In this article, we investigate the dynamics of the second messenger cAMP by performing stochastic simulations and parameter sweep analysis of a mechanistic model of the Ras/cAMP/PKA pathway, to determine the effects that the modulation of these feedback mechanisms has on the establishment of stable oscillatory regimes. In particular, we start by studying the role of phosphodiesterases, the enzymes that catalyze the degradation of cAMP, which represent the major negative feedback in this pathway. Then, we show the results on cAMP oscillations when perturbing the amount of protein Cdc25 coupled with the alteration of the intracellular ratio of the guanine nucleotides (GTP/GDP), which are known to regulate the switch of the GTPase Ras protein. This multi-level regulation of the amplitude and frequency of oscillations in the Ras/cAMP/PKA pathway might act as a fine tuning mechanism for the downstream targets of PKA, as also recently evidenced by some experimental investigations on the nucleocytoplasmic shuttling of the transcription factor Msn2 in yeast cells.

## Introduction

In living cells many processes are regulated by negative and positive feedback mechanisms, which are usually interlaced in complex regulatory networks and can function to either attenuate, amplify or even exploit molecular noise and stochasticity (see, e.g., [1-3] and references therein). As a matter of fact, molecular fluctuations do not always represent a negative feature for the proper functioning of a cellular system, on the contrary they can be advantageous to widen the range of stimulus-response to different perturbations, therefore promoting the adaptability to changeable environments. In this context, computational models represent an indispensable tool to investigate the complexity of the systems where multiple feedback and feedforward loops occur, multiple feedback and feedforward loops, as well as to reveal their emergent behaviors, as the use of experimental analysis alone is typically not able to unravel the whole picture of these (inhibitory or activatory) molecular interactions cascade [4-6].

A signal transduction pathway that is characterized by such complexity is the Ras/cAMP/PKA pathway in the yeast *Saccharomyces cerevisiae*, which regulates metabolism and cell cycle progression in response to nutritional sensing and stress conditions [7-10]. In budding yeast, five interlocked systems are known to participate in glucose signaling, which altogether result in a massive restructuring of the transcriptional state of the genome, as well as in a rapid change in the pattern of protein phosphorylation when glucose is added to cells growing on a non-fermentable carbon source [11]. Among these five pathways, the Ras/cAMP/PKA system plays a central role in responding to changes in glucose concentration and in turning on the processes that lead to cellular growth and division.

In particular, the Ras/cAMP/PKA pathway controls more than 90% of all genes that are regulated by glucose through the activation of the protein kinase A (PKA), that is able to phosphorylate a plethora of downstream proteins [11]. PKA is activated by the binding of the second messenger cyclic-AMP (cAMP), which is synthesized by the adenylate cyclase Cyr1. The activity of Cyr1 is controlled by the monomeric GTPases Ras1 and Ras2, which cycle between a GTP-bound active state and a GDP-bound inactive state. In turn, Ras proteins are positively regulated by protein Cdc25, a Ras-GEF (Guanine Nucleotide Exchange Factor) that stimulates the GDP to GTP exchange, and negatively regulated by proteins Ira1 and Ira2, two Ras-GAP (GTPase Activating Proteins) that stimulate the GTPase activity of Ras proteins. The degradation of cAMP is governed by two phosphodiesterases, Pde1 and Pde2. These two enzymes constitute a major negative feedback in this pathway: the low-affinity phosphodiesterase Pde1 is active under the positive regulation of PKA, while the high-affinity phosphodiesterase Pde2 is active in the basal level regulation of cAMP [7,12]. Experimental evidences suggest that the negative feedback loop exerted by PKA operates also at the level of Ras2-GTP [13-15]: PKA can phosphorylate Cdc25, reducing its exchange activity, as well as Ira proteins, increasing the Ras-GAP activity (in both cases, this regulation results in a decrease of the activity of the adenylate cyclase).

Because of such complex interplay, it is not easy to predict the behavior of the Ras/cAMP/PKA pathway in different growth conditions or in response to various stress signals. To understand the role of the negative feedback controls, in [16,17] we defined and analyzed a stochastic model of the Ras/cAMP/PKA pathway. In particular, we focused our attention on the mechanisms that allow the emergence of oscillatory regimes, since recent experiments evidenced *in vivo* the presence of continuous oscillations related to this pathway under specific stress conditions [18,19]. Furthermore, the effects of some regulatory mechanisms related to the stress response in the Ras/cAMP/PKA pathway were also highlighted through the analysis of the nucleocytoplasmic shuttling of Msn2, a transcription factor whose localization is controlled in yeast by the periodic activation of PKA [20,21]. This periodicity can be ascribed to an oscillatory behavior of the intracellular cAMP concentration and of PKA activity, though no direct measurements of the dynamics of these components have been executed *in vivo* so far. In this context, our previous computational investigations indicated that stable oscillatory regimes of cAMP amount can be established when the feedback operating on Ira proteins is activated, and that this dynamics seems to be regulated by the balance between the activities of the Ras protein modulators, i.e., Cdc25 and Ira proteins. In addition, we previously showed that also the intracellular ratio of guanine nucleotides pools (GTP/GDP) could represent an important metabolic signal for the regulation of the pathway, as also suggested in [22,23].

In this article, we extend the study presented in [24] and continue the analysis on the establishment of oscillatory regimes of cAMP by investigating the modulation of other feedback mechanisms. In particular, we study the influence that a change in the activity of phosphodiesterases - coupled with the perturbation of Cdc25 amount - have on the existence of stable oscillations of cAMP, and we highlight that the deletion of Pde1 can induce marked variations in the cAMP dynamics, while the deletion of Pde2 fosters the establishment of oscillations. Moreover, a preliminary analysis carried out on the oscillations frequency of cAMP in both the conditions of deletion of Pde1 and Pde2, considering different values for the ratio Cdc25/Ira2, shows that the deletion of Pde2 is able to diminish the oscillations frequency of cAMP with respect to the wild type condition, while the deletion of Pde1 has a minor effect on the frequency modulation.

Then, we continue the investigation initiated in [17] and study the role played by the guanine nucleotide concentrations, which control the exchange activity of Cdc25. Through the investigation of the simultaneous modulation of the amount of Cdc25 and of the intracellular ratio of guanine nucleotides, we show here that a decrease in the ratio GTP/GDP—which mimics a reduced nutritional condition in yeast cells—is able to control the transition between stable steady states and oscillations, independently from the amount of Cdc25.

## Methods

### Mechanistic model of the Ras/cAMP/PKA pathway

The mechanistic model of the Ras/cAMP/PKA pathway that we previously presented in [16,17] was developed according to the stochastic formulation of chemical kinetics [25], defined by specifying the set of molecular species occurring in the pathway and the set of biochemical reactions, together with their related stochastic constants (see Table 1). In particular, the model describes the major interactions between the pivotal components of the Ras/cAMP/PKA pathway, as well as the negative feedback mechanisms which are able to regulate the intracellular levels of cAMP. The model consists of six functional modules, which correspond to the following processes:

1. The switch cycle of Ras2 protein between its inactive state (Ras2-GDP) and active state (Ras2-GTP), regulated by the activity of the GEF Cdc25 and of the GAP Ira2 (reactions *r*_1_,…,*r*_10_ in Table 1).

2. The synthesis of cAMP through the activation of the adenylate cyclase Cyr1, mediated by Ras2-GTP (reactions *r*_11_,*r*_12_,*r*_13_ in Table 1).

3. The activation of PKA, mediated by the reversible binding of cAMP to its two regulatory subunits, and the subsequent dissociation of the PKA tetramer, which releases the two catalytic subunits (reactions *r*_14_,…,*r*_25_ in Table 1).

4. The activity of the two phosphodiesterases Pde1 and Pde2, that carry out the degradation of cAMP. The activation of Pde1 is regulated by the catalytic subunits of PKA, and it represents one of the main negative feedback control exerted by PKA within this pathway [12] (reactions *r*_26_,…,*r*_33_ in Table 1).

5. The negative feedback exerted by PKA on Cdc25, whose effect is modeled as a partial inactivation of the GEF activity, as stated in [15,26], and a reduction of the active state level of Ras2-GTP (reactions *r*_34_*r*_35_ in Table 1).

6. The negative feedback exerted by PKA on Ira2 which, according to [13,14], is assumed to increase the GAP activity and to induce a faster decrease of the Ras2-GTP level (reactions *r*_36_,…,*r*_39_ in Table 1).

**Table 1:** **Mechanistic model of the Ras/cAMP/PKA pathway in *S. cerevisiae ***

**No.**	**Reagents**	**Products**	**Constant *c***_***i***_
*r*_1_	Ras2-GDP + Cdc25	Ras2-GDP-Cdc25	1.0
*r*_2_	Ras2-GDP-Cdc25	Ras2-GDP + Cdc25	1.0
*r*_3_	Ras2-GDP-Cdc25	Ras2-Cdc25 + GDP	1.5
*r*_4_	Ras2-Cdc25 + GDP	Ras2-GDP-Cdc25	1.0
*r*_5_	Ras2-Cdc25 + GTP	Ras2-GTP-Cdc25	1.0
*r*_6_	Ras2-GTP-Cdc25	Ras2-Cdc25 + GTP	1.0
*r*_7_	Ras2-GTP-Cdc25	Ras2-GTP + Cdc25	1.0
*r*_8_	Ras2-GTP + Cdc25	Ras2-GTP-Cdc25	1.0
*r*_9_	Ras2-GTP + Ira2	Ras2-GTP-Ira2	^∗^1.0 ×10^−2^
*r*_10_	Ras2-GTP-Ira2	Ras2-GDP + Ira2	^∗^2.5 ×10^−1^
*r*_11_	Ras2-GTP + Cyr1	Ras2-GTP-Cyr1	1.0 ×10^−3^
*r*_12_	Ras2-GTP-Cyr1 + ATP	Ras2-GTP-Cyr1 + cAMP	2.1 ×10^−6^
*r*_13_	Ras2-GTP-Cyr1 + Ira2	Ras2-GDP + Cyr1 + Ira2	1.0 ×10^−3^
*r*_14_	cAMP + PKA	cAMP-PKA	1.0 ×10^−5^
*r*_15_	cAMP + cAMP-PKA	(2cAMP)-PKA	1.0 ×10^−5^
*r*_16_	cAMP + (2cAMP)-PKA	(3cAMP)-PKA	1.0 ×10^−5^
*r*_17_	cAMP + (3cAMP)-PKA	(4cAMP)-PKA	1.0 ×10^−5^
*r*_18_	(4cAMP)-PKA	cAMP + (3cAMP)-PKA	1.0 ×10^−1^
*r*_19_	(3cAMP)-PKA	cAMP + (2cAMP)-PKA	1.0 ×10^−1^
*r*_20_	(2cAMP)-PKA	cAMP + cAMP-PKA	1.0 ×10^−1^
*r*_21_	cAMP-PKA	cAMP + PKA	1.0 ×10^−1^
*r*_22_	(4cAMP)-PKA	C + C + R-2cAMP + R-2cAMP	1.0
*r*_23_	R-2cAMP	R + cAMP + cAMP	1.0
*r*_24_	R + C	R-C	7.5 ×10^−1^
*r*_25_	R-C + R-C	PKA	1.0
*r*_26_	C + Pde1	C + Pde1p	1.0 ×10^−6^
*r*_27_	cAMP + Pde1p	cAMP-Pde1p	1.0 ×10^−1^
*r*_28_	cAMP-Pde1p	cAMP + Pde1p	1.0 ×10^−1^
*r*_29_	cAMP-Pde1p	AMP + Pde1p	7.5
*r*_30_	Pde1p + PPA2	Pde1 + PPA2	1.0 ×10^−4^
*r*_31_	cAMP + Pde2	cAMP-Pde2	1.0 ×10^−4^
*r*_32_	cAMP-Pde2	cAMP + Pde2	1.0
*r*_33_	cAMP-Pde2	AMP + Pde2	1.7
*r*_34_	C + Cdc25	C + Cdc25p	1.0
*r*_35_	Cdc25p + PPA2	Cdc25 + PPA2	1.0 ×10^−2^
*r*_36_	Ira2 + C	Ira2p + C	1.0 ×10^−3^
*r*_37_	Ras2-GTP + Ira2p	Ras2-GTP-Ira2p	1.25
*r*_38_	Ras2-GTP-Ira2p	Ras2-GDP + Ira2p	2.5
*r*_39_	Ira2p	Ira2	10.0

The mechanistic model of the Ras/cAMP/PKA pathway in *S. cerevisiae*, developed according to the stochastic formulation of chemical kinetics, consists of 39 reactions among 33 molecular species. Each reaction is described by a set of reagents and a set of products, and is characterized by a stochastic constant ( *c*_*i*_, *i* = 1,…,39), here expressed in arbitrary time units (time^−1^). The following notation has been used in writing the reactions: (*i*) X + Y represents an interaction between the molecular species X and Y; (*ii*) X-Y describes a molecular complex between species X and Y; (*iii*) Xp denotes the phosphorylated form of species X; (*iv*) the regulatory and catalytic subunits of PKA are indicated by symbols R and C, respectively. Note that the values of the stochastic constants of reactions *r*_9_ and *r*_10_—highlighted with an asterisk in the table—are changed to 3.0×10^−2^ and 7.0×10^−1^, respectively, when reactions *r*_36_,…,*r*_39_ are not active, that is, when the negative feedback exerted by PKA on Ira2 is switched off.

**Figure 1:**
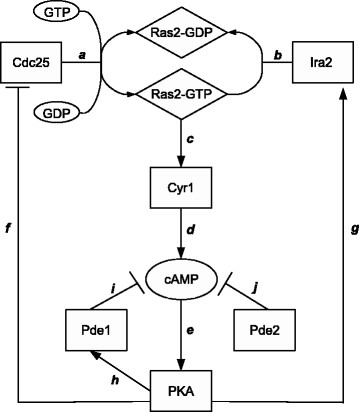
**Positive and negative regulations in the Ras/cAMP/PKA pathway.** The diagram shows the logical relationships among the principal components of the Ras/cAMP/PKA pathway. The switch cycle of Ras2 protein between its inactive state (Ras2-GDP) and active state (Ras2-GTP) is regulated by the activity of Cdc25 (**a**) and Ira2 (**b**). The intracellular ratio of GTP and GDP also contributes to the regulation of the activity of Ras proteins (**a**), since Cdc25 stimulates the exchange of these nucleotides on Ras according to their relative concentration. Ras2-GTP controls the activity of the adenylate cyclase Cyr1 (**c**), which mediates the synthesis of the second messenger cAMP (**d**). cAMP activates PKA (**e**) by binding to its regulatory subunits and releasing its catalytic subunits. The degradation of cAMP is governed by Pde1 (**i**) and Pde2 (**j**), which constitute a major negative feedback in this pathway, as they both contribute to decrease the intracellular level of the second messenger. The active form of PKA exerts three main regulations in this pathway through the phosphorylation of different components: a positive regulation of Pde1 (**h**) and of Ira2 (**g**), and a negative regulation of Cdc25 (**f**). Since the increased activity of the phosphorylated forms of both Pde1 and Ira2 result in switching off the signal—that is, they both contribute to reducing the intracellular level of cAMP—due either to a faster degradation of cAMP by Pde1 (**i**) or to a diminished fraction of active Ras2 by Ira2 proteins (**b**), these two positive regulations actually have the effect of a negative feedback control on the whole pathway. The negative regulation of Cdc25 by PKA results in a partial inactivation of the GEF activity (**a**), and thus a reduced activation of the Ras2 protein, which results in a decreased activity of the adenylate cyclase and therefore contributes to lowering the cAMP level.

In Figure 1, we give a schematic picture of the main inhibitory and activatory regulations existing among the components of the pathway. The complete network of the interactions between all molecular species, as well as the SBML version of the model, are available for free download at the BioSimWare website (http://biosimware.disco.unimib.it). A “generalized mass-action based” [27] version of this mechanistic model was derived, in order to compare the outcome of stochastic and deterministic approaches, as also discussed in [17].

Unless otherwise specified, all the simulations of our mechanistic model were performed starting from an initial state in which the Ras/cAMP/PKA pathway is switched off, that is, in a condition where no cAMP molecules are present in the system and the main components of the pathway (Ras2, adenylate cyclase, PKA) are inactive. The switch on of the pathway is triggered by the presence of an initial amount of the inactive form of Ras2 protein (Ras2-GDP complex), that can be transformed into the active form Ras2-GTP thanks to the presence of guanine nucleotide pools and of the Ras regulator proteins (the values of molecular species initially occurring in the system are given in Table 2). In cascade, the downstream components of the pathway are activated one after the other, giving rise to the emergent dynamics of the whole system and the resulting steady states. This situation is close to that observed *in vivo* when *S. cerevisiae* cells bearing a deletion in the GPR1 gene were starved for nutrients and then stimulated by glucose addition [28].

**Table 2:** Molecular amounts of initial species in the Ras/cAMP/PKA model

**Molecular species**	**Copy number (molecules/cell)**	**Reference**
Cyr1	200	[16]
Cdc25	300	[29]
Ira2	200	[16]
Pde1	1,400	[29]
PKA	2,500	[29]
PPA2	4,000	[29]
Pde2	6,500	[29]
Ras2-GDP	20,000	[29]
GDP	^∗^1.5 ×10^6^	[23]
GTP	^∗^5.0 ×10^6^	[23]
ATP	^∗^2.4 ×10^7^	[23]

The molecular amounts of the species initially occurring in the system are expressed as number of molecules per cell. The number of molecules for Ras2, Cdc25, PKA, Pde1, Pde2 and PPA2 were evaluated using the data presented in [29] (available online at http://yeastgfp.ucsf.edu/). The number of molecules for Ira2 and Cyr1 were estimated in [16] by comparing the fluorescence of yeast cells expressing fusion with eGFP (obtained by http://yeastgfp.ucsf.edu/) using Cdc25-eGFP as a standard (300 molecules/cell). The number of molecules for ATP, GTP and GDP were calculated by considering experimental data presented in [23] and assuming an internal free water volume of about 30 fL [16], obtained by considering an average cell volume of 45 fL [30,31] and taking into account that part of this volume is occupied by the cell wall and internal structures. Therefore, assuming a concentration of 1mM, for ATP we obtained about 2.4×10^7^ molecules/cell, while for GTP and GDP we estimated 5.0×10^6^ and 1.5×10^6^ molecules/cell, respectively, for yeast cells growing in minimal glucose medium [16]. Unless otherwise specified, the amounts of GDP, GTP and ATP—highlighted with an asterisk in the table—are kept constant during the execution of simulations.

The rationale behind this choice is that this initial condition allows us to investigate the transient accumulation as well as the oscillatory dynamics of cAMP according to a sequential activation of the different regulatory mechanisms within the pathway. To this aim, as also described in [16], the validation of the model was carried out by simulating the first functional module (the switch cycle of Ras2 protein) and then adding, in a sequential and iterative way, all the other modules of the model. So doing, we can easily identify the role played by every functional module of reactions on the emergent behaviors of the Ras/cAMP/PKA pathway, avoiding possible interferences with the molecular mechanisms that are already turned on in the system when starting the simulations from a different initial condition such as, e.g., a steady state corresponding to the basal level of cAMP. Nevertheless, we will show later on that the system response (e.g., the establishment of oscillatory regimes when the sixth functional module is activated) is actually independent of the chosen initial state of the system. For this reason, knowing that we obtain qualitatively and quantitatively comparable system responses starting from either a steady state condition or when the pathway is totally switched off, we prefer the latter initial state in order to analyze the pathway behaviors—in relation to both the initial transient and the subsequent dynamics in response to given stimuli—and to better compare the simulation outcomes under different perturbations.

### Simulation and analysis tools

The model was simulated and analyzed with the software BioSimWare [32], using a personal computer with an Intel Core2 CPU (2.66 GHz) running Linux. All stochastic simulations were performed by exploiting the tau-leaping algorithm [33], which represents one of the most efficient methods for simulating the temporal evolution of biochemical systems. This method is an approximated but accurate version of the stochastic simulation algorithm (SSA) defined in [25], which allows to select and execute in parallel several reactions per step—instead of executing the reactions in a sequential manner, as it is done with SSA—thus speeding up the computation. The mean duration time to execute one run of the tau-leaping algorithm to simulate the dynamics of the Ras/cAMP/PKA pathway over 1500 arbitrary time units is about 30 s, using the initial values of molecular amounts given in Table 2 and the stochastic constants reported in Table 1. Deterministic simulations were executed using the LSODA algorithm [34].

In this study, the efficiency of tau-leaping and LSODA algorithms was exploited to carry out a parameter sweep analysis (PSA), to the aim of investigating the effect of the variation of the values of molecular amounts and of reaction constants on the dynamics of cAMP and of other pivotal components of the Ras/cAMP/PKA pathway. PSA was performed using a computational tool that generates a set of different initial conditions for the model and then automatically executes the corresponding stochastic or deterministic simulations. With this tool, the value of each analyzed parameter varies within a specified range (with respect to a reference value), according to the following procedures: 

The sweep analysis for single parameters (PSA-1D) is performed considering a linear (logarithmic, respectively) sampling of values within the specified range in the case of molecular amounts (reaction constants, respectively). The logarithmic sampling allows to uniformly span different orders of magnitude of the value of the chosen parameter using a reduced but fine-grained set of samples, therefore efficiently analyzing the dynamics of the system in a broad range of environmental conditions.

The sweep analysis over pairs of parameters (PSA-2D) is performed by exploiting the quasi-random series method [35]. Quasi-random series, also called low discrepancy sequences, allow to efficiently sample a multidimensional space of numerical values. The discrepancy of a sequence represents a measure of its uniformity, and is computed by comparing the actual number of sampled points in a given multidimensional space with the number of points that would be sampled by assuming a uniform distribution. Therefore, the aim of quasi-random series is to uniformly cover the chosen parameter sweep space with “few” samples (i.e., with a lower number of points with respect to classic uniform distributions).

**Figure 2:**
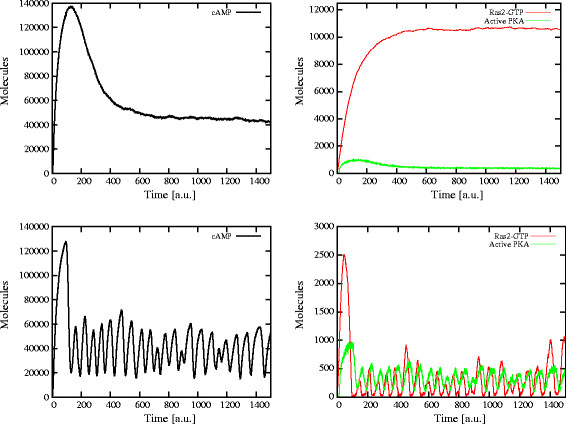
**Dynamics of cAMP, Ras2-GTP and active PKA with and without the feedback on Ira2.** When the feedback control on Ira2 proteins is not activated, the dynamics of cAMP shows an initial transient increase and the successive establishment of a stable steady state (*top left*). In this condition, neither Ras2-GTP nor active PKA show oscillatory behavior (*top right*). On the contrary, when the feedback on Ira2 proteins is activated, the initial peak on cAMP amount is followed by the establishment of a stable oscillatory state (*bottom left*), as also reflected in the dynamics of both Ras2-GTP and of the active fraction of PKA (*bottom right*). In *S. cerevisiae*, cAMP was experimentally determined to be around 2×10^5^ molecules after stimulation, while basal levels vary from 2×10^4^ to 5×10^4^ molecules/cell; as a consequence of stimulation, a cAMP peak was observed after 45–60 s, and then a new steady-state was reached in 3–5 min (see, e.g., Figure 2 in [28]). The expected number of cAMP molecules derived from stochastic simulations was calculated here by considering our own measurements [16] and data presented in [28].

Since we are interested in the analysis of the oscillatory regimes related to the Ras/cAMP/PKA pathway, we also developed a numerical procedure, implemented with the LabVIEW 2009 (National Instruments) environment, in order to evaluate the amplitude and frequency of stochastic oscillations. In particular, we considered the dynamics of cAMP as the target of this analysis. To this aim, for any simulation outcome we choose a portion of the dynamics where oscillations of cAMP occur (e.g., the time interval [200, 1,400] after the initial transient accumulation of cAMP in Figure 2, bottom left), we evaluate the mean amount of cAMP within this interval, and use this value as a threshold to identify the disjoint sets of consecutive points that are all above (or all below) the threshold. Then, within each of these sets, we identify the global maximum (or minimum, respectively) amount of cAMP, and finally we evaluate the mean and standard deviation of all the maxima (or minima) points previously identified. So doing, we can evaluate the maximum, minimum and average amplitude of the amount of cAMP during stochastic oscillations. The frequency of oscillations of cAMP can then be easily calculated by dividing the number of maxima (minima) by the length of the chosen time interval. We refer to [17] for additional details on this method.

## Results and discussion

The computational methods previously described were exploited in this study to test different hypotheses on the mechanisms that activate and regulate the components of the Ras/cAMP/PKA pathway in single yeast cells. In this context, our previous analysis on the Ras/cAMP/PKA model suggested that stable oscillatory regimes in the amount of cAMP can be regulated by the ratio between Cdc25 and Ira2 proteins, which both control the activation of the adenylate cyclase by means of the active fraction of Ras proteins (that is, Ras2-GTP) [17]. Hence, we start here by briefly presenting the effects of modulating the feedback mechanisms on Cdc25 and Ira2 proteins. Then, we study the role of the feedback mechanism exerted by PKA at the level of Pde1, as well as the influence of the deletion and of the overexpression of both phosphodiesterases on the establishment of oscillatory regimes. Finally, we investigate the presence of oscillations in the pathway through the variation of the intracellular amounts of GTP and Cdc25. Indeed, as we previously suggested [16], one of the signals that can modulate the activity of the Ras/cAMP/PKA pathway is the ratio between GTP and GDP, since the exchange activity of Cdc25 depends on the relative concentration of these guanine nucleotides [23].

### The role of feedback mechanisms on Ras activation

Starting from the initial condition of the system previously described, the simulation of the switch cycle of Ras2 protein—together with the activation of the adenylate cyclase and of the downstream components—shows a transient accumulation of cAMP in response to the formation of the complex Ras2-GTP. More precisely, when we only activate the feedback mechanisms based on the phosphorylation of Cdc25 and of the phosphodiesterase Pde1 (functional modules 1–5), we obtain a stable steady state in the levels of cAMP, Ras2-GTP and active PKA, as shown in Figure 2 (top plots). On the contrary, if the feedback control on Ira2 proteins is activated (functional module 6), then the system is able to generate stable oscillatory states of cAMP amount, as well as of Ras2-GTP and active PKA (Figure 2, bottom plots). We previously analyzed this oscillatory regime in [17], showing that the range of cAMP oscillations depends on the ratio between the Ras regulator proteins Cdc25 and Ira2, and that the oscillations frequency increases as the ratio Cdc25/Ira2 decreases, meaning that an unbalance between the GEF and GAP activity with respect to the wild type condition is able to induce a frequency modulation.

**Figure 3:**
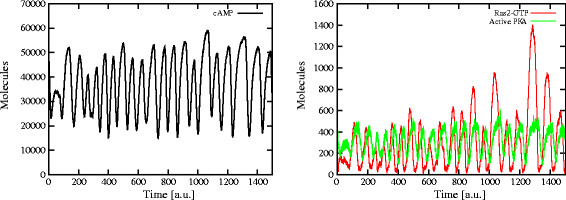
**Activation of the feedback on Ira2: cAMP oscillations starting from a steady state condition.** The occurrence of oscillatory regimes in the cAMP dynamics is only affected by the activation of negative feedback mechanisms and is independent of the initial condition of the system. The plots show the establishment of stable oscillations in cAMP amount (*left*), as well as in Ras2-GTP and active PKA amounts (*right*), starting the simulation from an initial condition where the system is at a stable steady state. In this simulation, the initial amounts of cAMP, Ras2-GTP and active PKA are around 47,000, 50, and 400 molecules, respectively.

In Figure 3, we show that the occurrence of the oscillatory regime in cAMP dynamics is affected only by the activation of these negative feedback mechanisms, and is actually independent of the chosen initial condition of the system. The plots show the establishment of stable oscillations in cAMP amount (left plot), as well as in Ras2-GTP and active PKA amounts (right plot), when the simulation is executed starting from an initial condition where the level of cAMP is already at the stable steady state.

**Figure 4:**
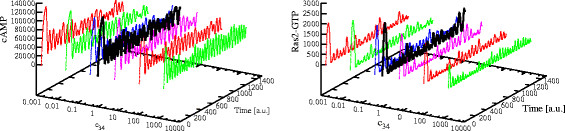
**Effects of the modulation of the negative feedback on Cdc25 (reaction constant*c***_**34**_**).** The figure shows the simulated dynamics of cAMP (*left*) and Ras2-GTP (*right*) resulting from a PSA-1D on the value of the reaction constant that modulates the phosphorylation of Cdc25 by means of active PKA. The varied parameter is constant *c*_34_ (see Table 1), within the interval [1.0×10^−3^, 1.0×10^3^], being 1.0 the reference value (represented with the black thick line). Stable oscillations occur in both cAMP and Ras2-GTP dynamics, for any value of the reaction constant, regardless of the magnitude of the feedback exerted by PKA on Cdc25. For larger values of the constant, namely *c*_34_ greater than 1.0, the only effect is a reduction in the amount of cAMP and Ras2-GTP (whose average values are reduced from around 40,000 to 35,000 molecules, and from around 270 to 130 molecules, respectively) and an increase of about fifty per cent in the frequency of oscillations with respect to the reference value.

**Figure 5:**
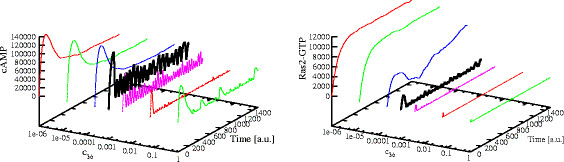
**Effects of the modulation of the negative feedback on Ira2 (reaction constant *c***_**36**_**).** The figure shows the simulated dynamics of cAMP (*left*) and Ras2-GTP (*right*) resulting from a PSA-1D on the value of the reaction constant that modulates the phosphorylation of Ira2 by means of active PKA. The varied parameter is constant *c*_36_(see Table 1), within the interval [1.0×10^−6^, 1.0], being 1.0×10^−3^ the reference value (represented with the black thick line). The reaction that describes the feedback on Ira2 is able to control the establishment of oscillatory regimes: for values of the reaction constant lower than the reference value, only stable steady states can be reached. In particular, cAMP and Ras2-GTP reach a noisy steady state around 60,000 and 10,000 molecules, respectively. Conversely, by increasing the value of the constant, that is, enhancing the GTPase activity of Ras2 proteins by means of the phosphorylation of Ira2, the amplitude of oscillations of cAMP and Ras2-GTP is reduced from around 40,000 to 25,000 molecules and from 600 to 140 molecules, respectively, as well as their average amounts, that decrease from around 40,000 to 20,000 and from around 270 to 50 molecules, respectively. For values of constant *c*_36_greater than 1.0×10^−2^ the oscillatory regime is lost and the average values of cAMP and Ras2-GTP drop to around 5,000 and 20 molecules, respectively.

Then, we investigated how an increased or a reduced phosphorylation activity of PKA over Cdc25 and Ira2 can influence the establishment of oscillatory regimes. Figures 4 and 5 represent the simulation results on the dynamics of cAMP (left plots) and of Ras2-GTP (right plots), carried out through a PSA-1D over the reaction constants corresponding to the negative feedback over Cdc25 and Ira2, respectively. Figure 4 shows that oscillations occur for any value of the stochastic constant of reaction *r*_34_, that is, regardless of the magnitude of the feedback exerted by PKA on Cdc25. On the contrary, the feedback on Ira2 is effectively able to control the establishment of oscillatory regimes (Figure 5): for values of the constant of reaction *r*_36_ lower than the reference value, only stable steady states can be reached. Conversely, if the value of this reaction constant is higher than the reference value, that is, if the GTPase activity of Ras2 proteins is strongly enhanced by the phosphorylation of Ira2, then the oscillatory regime is lost.

**Figure 6:**
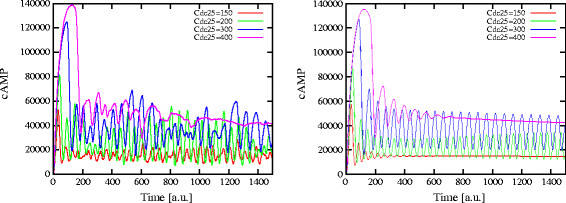
**Comparison of stochastic and deterministic simulations of cAMP dynamics with different initial amounts of Cdc25.** The figure shows the dynamics of cAMP with different initial amounts of Cdc25 (150, 200, 300, and 400 molecules) obtained by means of stochastic (*left*) and deterministic (*right*) simulations. The deterministic and stochastic behaviors are comparable for Cdc25 equal to 200 and 300 molecules (sustained oscillations occur in both cases) and for Cdc25 equal to 400 (damped oscillations). On the contrary, when the initial amount of Cdc25 is low (i.e., 150 molecules), with the stochastic approach we obtain stable oscillations of cAMP, while in the deterministic case the dynamics shows damped oscillations.

In addition, the stochastic simulations of the oscillatory regimes in the Ras/cAMP/PKA pathway were compared to the outcome of deterministic simulations of the “generalized mass-action model” [17]. In Figure 6, we show the dynamics of cAMP with different initial amounts of Cdc25, obtained by means of stochastic (left plot) and deterministic (right plot) simulations. It is worth noting that the deterministic and stochastic behaviors are comparable for Cdc25 equal to 200 and 300 molecules (sustained oscillations occur in both cases) and for Cdc25 equal to 400 (damped oscillations). On the other hand, by setting the initial amount of Cdc25 to smaller values, e.g., 150 molecules, the two approaches show qualitatively different outcomes: the stochastic approach provides stable oscillations of cAMP, while in the deterministic case, under the same initial conditions, the dynamics show damped oscillations. This result highlights the usefulness of stochastic modeling and the role played by noise in the Ras/cAMP/PKA pathway, which seems to support the robustness of the system with respect to the variation of the amount of pivotal components of the pathway (in this case, protein Cdc25), ensuring the presence of stable oscillatory regimes.

### The negative regulation by phosphodiesterases

**Figure 7:**
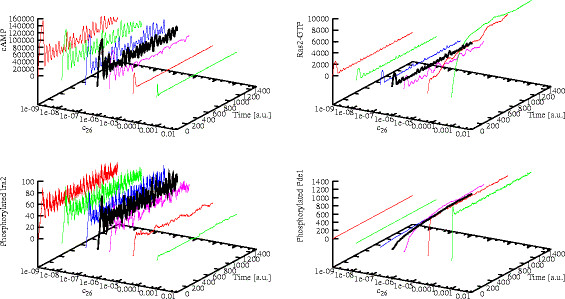
**Effects of the modulation of the negative feedback on Pde1 (reaction constant *c***_**26**_**).** The figure shows the simulated dynamics of cAMP (*top left*), Ras2-GTP (*top right*), phosphorylated Ira2 (*bottom left*) and phosphorylated Pde1 (*bottom right*) resulting from a PSA-1D on the value of the reaction constant that modulates the phosphorylation of Pde1 by means of active PKA. The varied parameter is constant *c*_26_ (see Table 1), within the interval [1.0×10^−9^, 1.0×10^−3^], being 1.0×10^−6^ the reference value (represented with the black thick line). For values of the constant lower than the reference value, stable oscillations still occur. On the contrary, by increasing the value of the reaction constant, that is, if we simulate a marked promotion of the activity of Pde1, after an initial transient increase the amount of cAMP gets almost completely degraded and no oscillations occur anymore (cAMP reaches a noisy steady state around 5,000 molecules, *top left*). Under the same condition, the amount of Ras2-GTP tends to a high steady state level (around 10,000 molecules, *top right*), which would intuitively induce a promotion of the cyclase activity and thus an expectable increase in the cAMP amount, which is instead counterbalanced by two concurrent processes: (i) the reduced activity of Ira2 proteins, whereby only a few copies of phosphorylated Ira2 are present inside the system (around 5 molecules, *bottom left*), and (ii) the strong phosphodiesterase activity taking place in this condition, that is also confirmed by the high level of phosphorylated Pde1 (around 1,200 molecules, *bottom right*).

**Figure 8:**
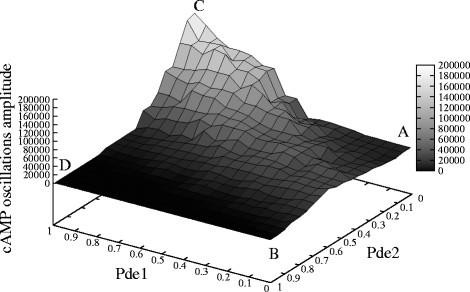
**Influence of the amount of Pde1 and Pde2 on the establishment of cAMP oscillatory regimes.** The figure shows the oscillations amplitude of cAMP dynamics resulting from a PSA-2D on the values of the initial amounts of Pde1 and Pde2, varied in the intervals [0, 2, 800] and [0, 13, 000], respectively (being Pde1 = 1,400 and Pde2 = 6,500 molecules the reference values—see Table 2). In the plot, the values on the *x*- and *y*-axis were normalized in the interval [0, 1]; a total of 200 initial conditions were sampled from the specified bidimensional parameters space. This analysis shows that the deletion of the high-affinity phosphodiesterase Pde2 fosters the establishment of oscillations of cAMP, whose amplitude increases with the increase of Pde1 amount (from point **A** to point **C**); on the contrary, the deletion of the low-affinity cAMP phosphodiesterase Pde1 has the effect of diminishing or even abolishing the oscillations irrespective of the amount of Pde2 (from point **A** to **B**). The overexpression of both phosphodiesterases (point **D**) does not allow the establishment of oscillatory regimes.

**Figure 9:**
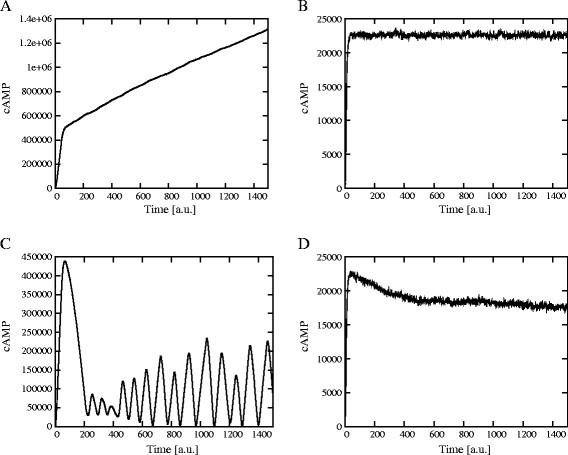
**cAMP dynamics obtained with different initial amounts of Pde1 and Pde2.** Dynamics of cAMP with different initial amounts of the phosphodiesterases, as given in Figure 8, where **A**: Pde1 = 0, Pde2 = 0; **B**: Pde1 = 0, Pde2 = 13,000; **C**: Pde1 = 2,800, Pde2 = 0; **D**: Pde1 = 2,800, Pde2 = 13,000 molecules. The plots show that in the absence of both phosphodiesterases (**A**) we achieve, as expected, an unlimited accumulation of cAMP, since our model does not include any other mechanism to reduce the intracellular level of cAMP; with a very high initial amount of Pde2 (**B** and **D**), cAMP reaches a noisy steady state but no oscillations are observed; in the absence of Pde2, when only Pde1 is active (**C**), oscillations of cAMP can then be established, showing a very large amplitude and a mean cAMP amount that is slightly higher than standard conditions. The molecular amounts of cAMP reached in conditions **A** and **C** are higher than the physiological levels measured in corresponding experimental settings [12,14] though, from a computational point of view, they are indicative of the role played by the two phosphodiesterases, since they highlight the different dynamical behaviors of the pathway in extreme conditions.

**Figure 10:**
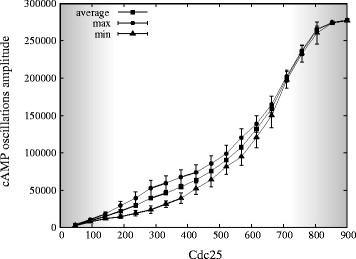
**Diagram of cAMP oscillations amplitude with respect to Cdc25 amount when deleting Pde1.** The figure shows the oscillations amplitude of cAMP dynamics resulting from a PSA-1D where the amount of Cdc25 varies in the interval [0, 900], in the condition of deletion of Pde1. The mean and standard deviation of the average (squares), maximum (circles) and minimum (triangles) amount of cAMP during oscillations are plotted. The left and right shady areas correspond to noisy stochastic fluctuations and stable steady states, respectively, while the white area corresponds to oscillatory regimes. This analysis highlights that, when deleting Pde1, oscillatory regimes in cAMP can be established even when the amount of Cdc25 is at a two-fold overexpression with respect to the physiological amount of 300 molecules/cell (Table 2). Therefore, the deletion of Pde1 has the effect of widening the range of Cdc25 molecules under which sustained oscillations of cAMP occur, being approximately [150, 400] the interval whereby oscillatory regimes in cAMP are found when Pde1 is present in the system (see [17] for more details).

**Figure 11:**
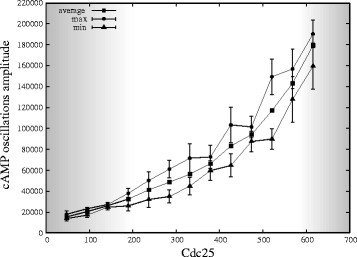
**Diagram of cAMP oscillations amplitude with respect to Cdc25 amount when deleting Pde2.** The figure shows the oscillations amplitude of cAMP dynamics resulting from a PSA-1D where the amount of Cdc25 varies in the interval [0, 900], in the condition of deletion of Pde2. The mean and standard deviation of the average (squares), maximum (circles) and minimum (triangles) amount of cAMP during oscillations are plotted. The left and right shady areas correspond to noisy stochastic fluctuations and stable steady states, respectively, while the white area corresponds to oscillatory regimes. Similarly to Figure 10, the analysis highlights that, under the deletion of Pde2, oscillatory regimes in cAMP can be established even when the amount of Cdc25 is at a two-fold overexpression with respect to its physiological amount. Therefore, also the deletion of Pde2 has the effect of changing the range of Cdc25 molecules under which sustained oscillations of cAMP occur.

To determine the influence of phosphodiesterases on the existence of stable oscillations of cAMP, we conducted three different parameter sweep analyses: 

1. A PSA-1D over the reaction constant corresponding to the negative feedback exerted by PKA on Pde1, whereby higher values of this parameter represent a stronger activation of the phosphodiesterase activity, and hence a higher net effect of the negative feedback. In Figure 7, we plot the dynamics of cAMP (top left plot), Ras2-GTP (top right plot), phosphorylated Ira2 (bottom left plot) and phosphorylated Pde1 (bottom right plot) with respect to the variation of this reaction constant in the interval [1.0×10^−9^, 1.0×10^−3^]. This interval corresponds to 3 orders of magnitude below and 3 above the reference value given in Table 1, whose related dynamics is represented in the plots with the black thick line.

2. A PSA-2D over the amounts of Pde1 and of Pde2 in the intervals [0, 2,800] and [0, 13,000] molecules, respectively, which mimic the biological conditions ranging from the deletion to a two-fold overexpression of each phosphodiesterase. In Figure 8 we plot the amplitude of cAMP oscillations generated with these parameters, where the values on the *x*- and *y*-axis were normalized to [0, 1]. In this figure, an amplitude value equal to zero corresponds to a non oscillating dynamics. Figure 9 shows the dynamics of cAMP in the four extreme conditions of the phosphodiesterases amounts, as highlighted in Figure 8, where **A** corresponds to Pde1 = 0, Pde2 = 0 molecules; **B** corresponds to Pde1 = 0, Pde2 = 13,000 molecules; **C** corresponds to Pde1 = 2,800, Pde2 = 0 molecules; **D** corresponds to Pde1 = 2,800, Pde2 = 13,000 molecules.

3. A PSA-1D over the amount of Cdc25 in the interval [0, 900] molecules, ranging from the deletion to a three-fold overexpression of the GEF proteins, in both conditions of deletion of Pde1 or Pde2. In Figures 10 and 11, we plot the diagrams of the amplitude of cAMP oscillations with respect to the number of Cdc25 molecules, under the deletion of Pde1 and Pde2, respectively. In these figures, square points represent the mean value of cAMP amount, circle (triangular) points the maximum (minimum) value of oscillations with the respective standard deviation, the left and right shady areas correspond to noisy stochastic fluctuations and stable steady states, respectively, while the white area corresponds to oscillatory regimes.

Taken altogether, the results of these simulations show that the deletion of the high-affinity phosphodiesterase Pde2 fosters the establishment of oscillations of cAMP, whose amplitude increases with the increase of Pde1 amount. On the contrary, the deletion of the low-affinity cAMP phosphodiesterase Pde1 has the effect of diminishing or even abolishing the oscillations irrespective of the amount of Pde2 (see Figure 8). This might indicate that the negative feedback on Pde1 is effectively able to regulate the oscillatory regime of cAMP, independently from the presence of Pde2.

Indeed, if we simulate a stronger activity of PKA over Pde1, that is, a marked promotion of the activity of Pde1, we see that after an initial transient increase the intracellular cAMP gets almost completely degraded and no oscillations occur anymore (Figure 7, top left plot). Interestingly, in the same condition the amount of Ras2-GTP tends to a high steady state level (Figure 7, top right plot), which would intuitively induce a promotion of the cyclase activity and thus an increase in the cAMP amount. This counterintuitive behavior is an overall effect due to two concurrent factors: (*i*) the strong negative feedback exerted by Pde1 (Figure 7, bottom right plot), that causes the immediate degradation of cAMP and does not allow its intracellular accumulation, and (*ii*) the lack of the effect of the feedback on Ira2, that causes the increase in the amount of Ras2-GTP, a consequence of the fact that Ira2 proteins are basically not phosphorylated by PKA in this condition (Figure 7, bottom left plot).

In addition, Figure 10 shows that in the absence of Pde1 the oscillatory regimes are established even when the amount of Cdc25 is at a two-fold overexpression with respect to its physiological amount, which corresponds to about 300 molecules/cell [29]. These data can be compared to the analysis shown in [17], which highlights that in normal conditions and in presence of Pde1, the oscillatory regimes can only be established when Cdc25 is approximately between 150 and 400 molecules, that is, when the ratio Cdc25/Ira2 is not higher than 2 (being the amount of Ira2 around 200 molecules in normal conditions). Therefore, the deletion of Pde1 with respect to the ratio Cdc25/Ira2 has the effect of widening the conditions under which sustained oscillations of cAMP occur. Similar considerations can be done for the deletion of Pde2, whereby oscillatory regimes occur with Cdc25 in between 200 and 600 molecules (Figure 11).

The computational results corresponding to the conditions of deletion of the phosphodiesterases are in line with recent experimental measurements of the nucleocytoplasmic localization of the transcription factor Msn2, carried out in *S. cerevisiaepde1Δ* and *pde2Δ* mutant cells, under continuous light-induced stress conditions [21]. In yeast cells, the nuclear localization of Msn2 is under the negative control of PKA: it is mainly localized in the cytoplasm under non-stressed conditions, but in response to environmental stresses Msn2 is dephosphorylated and translocates to the nucleus. The observations presented in [21] highlight that both deletion mutants—that are characterized by a higher PKA activity with respect to the control strain—show a decrease in Msn2 nuclear localization, with *pde2Δ* exhibiting the strongest effect, that is, a marked reduction of nuclear Msn2 with respect to both *pde1Δ* and the control strain. Moreover, both phosphodiesterases seem to be involved in the regulation of cAMP intracellular amount under light-induced stress. Indeed, in cells lacking the phosphodiesterases, the PKA activity was shown to increase, in agreement to our simulation outcomes, therefore inducing a decrease in the nuclear fraction of Msn2 [21].

**Figure 12:**
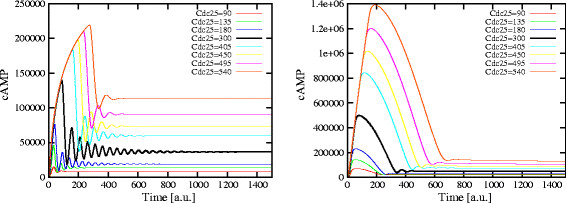
**Deterministic simulations of cAMP dynamics under deletion of Pde1 or Pde2 with different initial amounts of Cdc25.** The figure shows the dynamics of cAMP with different initial amounts of Cdc25, obtained by means of deterministic simulations when deleting Pde1 (*left*) and Pde2 (*right*). In both conditions, no sustained oscillations are present in the system for any value of the initial amount of Cdc25.

**Figure 13:**
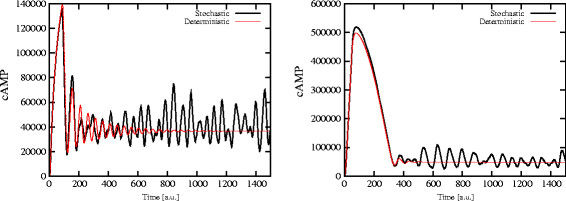
**Comparison of stochastic and deterministic simulations of cAMP dynamics under deletion of Pde1 or Pde2, with Cdc25 = 300 molecules.** The figure shows the comparison of the dynamics of cAMP obtained by means of stochastic and deterministic simulations when deleting Pde1 (*left*) or Pde2 (*right*), with an initial amount of Cdc25 equal to 300 molecules. We note that, while in the deterministic case oscillations are damped or even not occurring, the stochastic case shows sustained oscillations of cAMP in both cases.

Finally, an analysis similar to that presented in Figures 10 and 11 was performed for the deterministic case, though achieving different results with respect to the stochastic approach. As shown in Figure 12, which represents the dynamics of cAMP under different initial amounts of Cdc25, in both cases of deletion of Pde1 (left plot) and Pde2 (right plot) no sustained oscillations of cAMP are obtained (for this reason, the diagram of oscillations amplitude corresponding to the deterministic simulations—as given in Figures 10 and 11 in relation to the stochastic approach—is not shown, as it would be non informative). Instead, in Figure 13 we show the comparison between the dynamics of cAMP obtained with stochastic and deterministic simulations, under the deletion of Pde1 (left plot) and Pde2 (right plot), and with an initial amount of Cdc25 equal to 300 molecules, which represents, the physiological level in yeast cells (Table 2). The plots clearly show that in both conditions, while with the deterministic approach oscillations are damped or even not occurring, stochastic simulations show sustained oscillations of cAMP. Therefore, we can hypothesize that the introduction of noise in the Ras/cAMP/PKA pathway is able to stabilize the oscillatory regimes.

**Figure 14:**
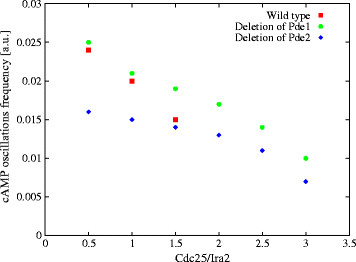
**Frequency modulation of cAMP oscillations in the wild type condition and under the deletion of Pde1 and Pde2.** The figure shows the comparison of the oscillations frequency of cAMP in the wild type condition (red square dots) and under the deletion of Pde1 (green circle dots) or Pde2 (blue diamond dots), with respect to the ratio Cdc25/Ira2 (with the initial amount of Cdc25 ranging from 100 to 600 molecules, and Ira2 = 200 molecules). The plot shows that the deletion of Pde1 has a minor effect with respect to the wild type condition on the modulation of frequency, while the deletion of Pde2 is able to diminish the oscillations frequency of cAMP. For values of the ratio Cdc25/Ira2 greater than 1.5, the points related to the wild type condition are not plotted, since no stable oscillations are established in these cases [17].

In this context, we also carried out a preliminary analysis on the oscillations frequency of cAMP in both the conditions of deletion of Pde1 and Pde2, considering different values for the ratio Cdc25/Ira2, as already mentioned in the previous section for the wild type condition. In Figure 14, we compare the oscillations frequency of cAMP in these three conditions showing that, while the deletion of Pde1 has a minor effect with respect to the wild type on the frequency modulation, the deletion of Pde2 is able to diminish the oscillations frequency of cAMP, as can also be gained by comparing the stochastic simulations of cAMP dynamics presented in Figure 13 and in Figure 2, bottom left.

### The role of guanine nucleotide pools

We previously suggested that one of the signals able to modulate the activity of the Ras/cAMP/PKA pathway is the ratio between GTP and GDP, since the exchange activity of Cdc25 depends on the relative concentration of GTP and GDP [16,23]. In normal growth conditions, the concentration of GTP is 3 to 5 times higher than GDP, allowing the activation of Ras protein; anyway, under limited nutrient availability (when the relative amount of GTP decreases), the activity of Cdc25 does not result in Ras proteins activation, since in this case the unproductive binding/unbinding with GDP is mostly favored.

**Figure 15:**
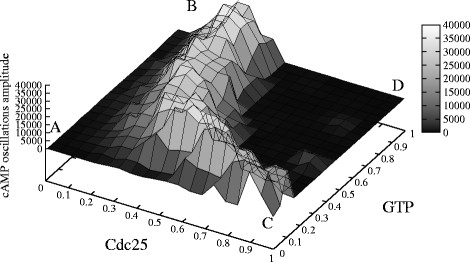
**Influence of the amount of GTP and Cdc25 on the establishment of cAMP oscillatory regimes.** The figure shows the oscillations amplitude of cAMP dynamics resulting from a PSA-2D on the values of the initial amounts of GTP and Cdc25, varied in the intervals [1.9×10^4^, 5.0×10^6^] and [0,600], respectively (being GTP = 5.0×10^6^ and Cdc25 = 300 molecules the reference values—see Table 2). In the plot, the values on the *x*- and *y*-axis have been normalized in the interval [0, 1]; a total of 200 initial conditions were sampled from the specified bidimensional parameters space. This analysis shows that when the amount of Cdc25 is approximately at normal condition or slightly lower, oscillatory regimes are established for basically any value of GTP (from point **A** to point **B**), being the amplitude of oscillations smaller in lower nutrient availability conditions. On the contrary, when the amount of Cdc25 increases, no oscillations of cAMP occur when GTP is high (point **D**), but oscillatory regimes are still present if GTP is low (point **C**).

**Figure 16:**
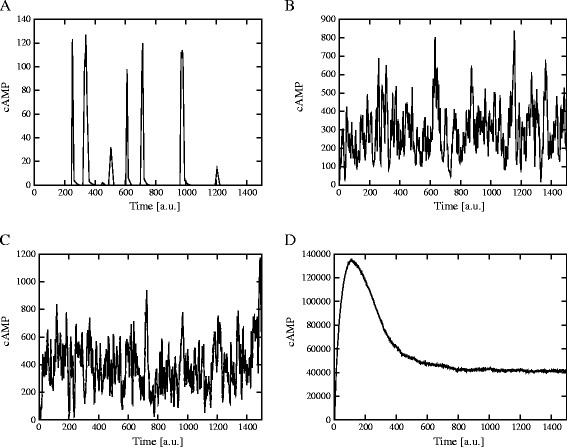
**cAMP dynamics obtained with different initial amounts of Cdc25 and GTP.** The figure shows the dynamics of cAMP with different initial amounts of Cdc25 and GTP, as given in Figure 15, where **A**: Cdc25 = 10, GTP = 1.9×10^4^; **B**: Cdc25 = 10, GTP = 5.0×10^6^; **C**: Cdc25 = 600, GTP = 1.9×10^4^; **D**: Cdc25 = 600, GTP = 5.0×10^6^ molecules. The plots show that low amounts of Cdc25 (**A** and **B**), or high amounts of Cdc25 coupled with low amounts of GTP (**C**), lead to the production of very low amounts of cAMP, showing a noisy dynamics. On the other hand, when the amounts of both Cdc25 and GTP are high (**D**), we observe a dynamics of cAMP similar to that obtained without the feedback on Ira2 proteins (see Figure 2, top left), and no oscillations are established.

To investigate the role played by guanine nucleotides concentrations on the establishment of oscillations, we carried out a PSA-2D to simulate the behavior of the system in perturbed conditions, where the concentration of GTP varies in the interval [1.9×10^4^, 5.0×10^6^] molecules (ranging from a reduced nutrient availability to a normal growth condition) and, at the same time, also the amount of Cdc25 varies in the interval [0,600] molecules (ranging from the deletion to a two-fold overexpression of the GEF proteins). In Figure 15, we plot the amplitude of cAMP oscillations obtained in these conditions. In this figure, the values on the *x*- and *y*-axis were normalized to [0, 1], and an amplitude value equal to zero corresponds to a non oscillating dynamics. Figure 16 shows the dynamics of cAMP in the four extreme conditions of GTP and Cdc25 amounts, as highlighted in Figure 15, where **A** corresponds to Cdc25 = 10, GTP = 1.9×10^4^ molecules; **B** corresponds to Cdc25 = 10, GTP = 5.0×10^6^ molecules; **C** corresponds to Cdc25 = 600, GTP = 1.9×10^4^ molecules; **D** corresponds to Cdc25 = 600, GTP = 5.0×10^6^ molecules.

The simulations show that when the amount of Cdc25 is approximately at normal condition or slightly lower, the oscillatory regimes are established for basically any value of GTP, being the amplitude of oscillations smaller in lower nutrient availability conditions. On the contrary, when the amount of Cdc25 increases, no oscillations of cAMP occur when GTP is high, but oscillatory regimes are still present if GTP is low. This result can be motivated considering that when the ratio GTP/GDP decreases, Ras proteins are more frequently loaded with GDP instead that with GTP, and their activity is therefore decreased, inducing the establishment of an oscillatory regime.

## Conclusion

With this study we determined, in a quantitative way, that the coupling between feedback mechanisms and the molecular levels of the Ras modulators can influence the oscillatory regimes of cAMP and PKA. In this context, our study highlights the role played by the feedback exerted by PKA on phosphodiesterases and on Ira2 proteins, that was never directly investigated so far. To this aim, stochastic and deterministic simulations were carried out to analyze the behavior of the Ras/cAMP/PKA pathway under different conditions. As also presented in [17], the comparison between the two approaches indicates that with deterministic simulations the interval of Cdc25 amount for obtaining stable oscillations of cAMP is reduced with respect to stochastic simulations. In particular, in [17] it was shown that stable oscillations occur when Cdc25 amount is approximately between 200 and 350 molecules in the first case, while in the second case noisy oscillations are still evident for lower and higher Cdc25 amounts (being the oscillatory regime interval around [150, 400] molecules). Within this oscillatory interval, the frequency and the amplitude of oscillations are well comparable in the stochastic and the deterministic simulations in standard conditions.

On the contrary, the comparison between stochastic and deterministic analysis performed in the perturbed conditions (that is, under the deletion of phosphodiesterases) shows qualitatively and quantitatively different results. Indeed, the dynamics of cAMP with different initial amounts of Cdc25, in both cases of deletion of Pde1 and Pde2, does not present sustained oscillations in the deterministic case, while stochastic simulations show stable oscillations.

Therefore, we can argue that molecular noise within the Ras/cAMP/PKA pathway can enhance the robustness of the system at least in response to the different perturbations we considered here, ensuring the presence of stable oscillatory regimes as also previously discussed for other biological systems (see [2] and references therein). Indeed, stochastic simulations show that the cell might be able to respond appropriately to an alteration of its pivotal components—such as the amount of protein Cdc25, which is related to the stress level [19,36]—fostering the maintenance of stable oscillations during the signal propagation (i.e., the synthesis of the second messenger cAMP) and the activation of PKA. As such, this might suggest a stronger adaptation capability of yeast cells to various environmental stimuli or endogenous variations.

In [37] it was shown that in MIN6 beta cells PKA, cAMP and calcium are highly integrated in an oscillatory circuit that allows a fine spatiotemporal regulation of the kinase activity. Similarly, we think that the multi-level regulation carried out with different feedback mechanisms in the Ras/cAMP/PKA pathway in yeast might represent a way to extend the regulatory span of the system, therefore acting as a tuning mechanism for the numerous downstream targets of PKA. This assumption might be in line with the hypothesis of the “frequency-modulated” regulation that was recently proposed in yeast in relation to calcium oscillations [38], though further computational and experimental investigations should be carried out to ascertain the validity of this hypothesis also in relation to cAMP and PKA oscillations induced by the molecular interactions within the Ras/cAMP/PKA pathway.

Indeed, oscillations related to the Ras/cAMP/PKA pathway were experimentally observed, but only in indirect ways, e.g., through the analysis of the periodic nucleocytoplasmic shuttling of Msn2 [18,19]. In this context, the observations presented in [21] show that, in single yeast cells subject to continuous light exposure, the oscillations frequency of Msn2 between the nucleus and the cytoplasm can be influenced by PKA as well as by Pde1 and Pde2, whereby phosphodiesterases indirectly affect the activity of PKA through the degradation of cAMP. In particular, in [21] it was shown that the oscillation frequency of Msn2 increases alongside the increase in the induced-stress condition, which can be also ascribed to a reduced activity of Cdc25 protein [19,36], as tested in our study. Anyway, it is not clear whether the nucleocytoplasmic oscillations frequency of Msn2 can be interpreted solely in terms of the above mentioned frequency modulation control, since the response of the cell to cumulative light-induced stress might suggest a more complex scenario that is still to be unraveled [21].

The computational results presented in this study and in [17], in relation to the amplitude and the frequency of oscillations within the Ras/cAMP/PKA pathway, suggest that a frequency modulation can be achieved when perturbing the ratio between the amounts of Cdc25 and Ira2 proteins, that is, the Ras regulator proteins. In particular we showed here that, with respect to the wild type condition, the deletion of Pde2 is able to diminish the oscillations frequency of cAMP, while the deletion of Pde1 has a minor effect on its variation. These results represent a first step towards an in depth analysis of oscillatory regimes in the Ras/cAMP/PKA pathway, that we plan to carry out in different ways. On the one hand, we will investigate the correlation between the oscillations of cAMP, PKA and its downstream targets (such as Msn2) to analyze how this behavior propagates through the signal transduction pathway. On the other hand, we are currently developing a computational tool to quantitatively characterize the oscillations (whether stochastic, deterministic or noise-induced) by means of Fourier analysis, as already proposed for the study of the oscillatory shuttling of NF-*κ*B [39], whereby the power spectrum of simulated dynamics was analyzed to verify the occurrence of peaks at non-zero frequencies, as well as to calculate the signal-to-noise ratio, in order to inspect the presence of oscillations. In addition, qualitative analysis of the nature of bifurcation points, based on dynamical systems theory (see, e.g., [40] for an application to the study of noise-induced stabilization in a genetic circuit, and [41] for a broad overview of the subject), could be exploited to better investigate how stochastic fluctuations are able to originate the stable oscillatory regimes in the Ras/cAMP/PKA model, which do not occur in the absence of noise.

Furthermore, as a future development of our study, we will investigate the response of the Ras/cAMP/PKA pathway to nutrients and to intracellular acidification (that likely causes an inhibition of GAP activity of the Ira proteins [42]), its crosstalk and integration with other pathways co-involved in glucose signaling and yeast metabolism, as well as the regulated expression of downstream target genes. In particular, we will define additional functional modules of reactions to describe the Gpr1/Gpa2 pathway, a signaling mechanism that responds only to high glucose concentration and operates in an addictive redundant way with Ras2-GTP to activate the adenylate cyclase [28,42]. We also plan to define a multi-volume version of our mechanistic model, in order to characterize the intracellular localization of the central components of this pathway, since there exist experimental evidences that most of the Cdc25, Cyr1, Ira2 and Ras2 proteins localize at internal membranes, suggesting the presence of large signaling complexes inside yeast cells [43,44]. Investigations about the topological distribution of the molecular species in distinct cellular regions will be performed by means of the tau-DPP framework [45]. This will enable us, for instance, to study the dynamical movement of Cdc25 proteins to plasma membrane in response to nutrient starvation, and the hyper-activity of PKA to counteract the localization of Cdc25 and Ira2 proteins.

In conclusion, the computational model we developed allows to investigate in details the mechanisms that regulate the transition between stable and oscillatory regimes in the Ras/cAMP/PKA pathway, to make predictions on the conditions that lead to the insurgence of oscillations, and to eventually plan focused validation experiments. In particular, by directly operating on the modulation of specific components of the pathway, such as molecular amounts and reaction constants, we are able to study in details the influence of every molecular interaction on the pathway behavior. Nonetheless, although the Ras/cAMP/PKA pathway has gone through extensive investigations in *S. cerevisiae*, accurate wet data on the spatiotemporal dynamics of cAMP in single yeast cells are still lacking. To this aim, we are carrying out extensive laboratory work to develop a FRET-sensor based on Epac able to respond to cAMP levels in *S. cerevisiae*, in order to measure the changes in the level of cAMP in single cells and to directly test the presence of long term cAMP oscillations *in vivo*[46]. This setup will allow us to conduct an in depth analysis of the response of the pathway to different nutritional and stress conditions, as well as to perform an accurate parameter estimation analysis [47], therefore working thoroughly on the experimental and computational validation of our model.

## Competing interests

The authors declare that they have no competing interests.
